# Remaining Useful Life Prediction of Airplane Engine Based on PCA–BLSTM

**DOI:** 10.3390/s20164537

**Published:** 2020-08-13

**Authors:** Shixin Ji, Xuehao Han, Yichun Hou, Yong Song, Qingfu Du

**Affiliations:** School of Mechanical, Electrical & Information Engineering, Shandong University, Weihai 264209, China; jishixin@mail.sdu.edu.cn (S.J.); 17763156222@163.com (X.H.); nipperhou@mail.sdu.edu.cn (Y.H.); songyong@sdu.edu.cn (Y.S.)

**Keywords:** airplane engine, principal component analysis, bidirectional long short-term memory, prediction of remaining useful life

## Abstract

The accurate prediction of airplane engine failure can provide a reasonable decision basis for airplane engine maintenance, effectively reducing maintenance costs and reducing the incidence of failure. According to the characteristics of the monitoring data of airplane engine sensors, this work proposed a remaining useful life (RUL) prediction model based on principal component analysis and bidirectional long short-term memory. Principal component analysis is used for feature extraction to remove useless information and noise. After this, bidirectional long short-term memory is used to learn the relationship between the state monitoring data and remaining useful life. This work includes data preprocessing, the construction of a hybrid model, the use of the NASA’s Commercial Aerodynamic System Simulation (C-MAPSS) data set for training and testing, and the comparison of results with those of support vector regression, long short-term memory and bidirectional long short-term memory models. The hybrid model shows better prediction accuracy and performance, which can provide a basis for formulating a reasonable airplane engine health management plan.

## 1. Introduction

With the development of an airplane engine towards integration, systematization and precision, the engine system becomes more and more complex. In order to prevent the occurrence of failure, it is necessary to invest in high maintenance and servicing costs. In addition, many faults of airplane engines occur randomly, so it is difficult to predict the time of faults according to previous experience, resulting in that many faults cannot be prevented by taking measures in advance. At the beginning of this century, American domestic companies spent nearly a trillion dollars a year to maintain major systems, of which one-third to one-half of the expenditure was lost due to invalid maintenance [1]. In general, the serious faults of complex equipment do not occur suddenly, but are evolved step-by-step from the gradual incipient faults phase. If we can the detect problems in a timely manner when the system is still in gradual incipient faults, we can take the measures to maintain it in advance to avoid major breakdowns due to continued development, which is of great significance for the maintenance of equipment. Therefore, according to the needs of airplane health management, the status monitoring data of an airplane were used to predict the remaining useful life (RUL) of airplane engines. According to the predicted results, targeted pre-maintenance plans are made to provide a basis for airplane engine maintenance and maintenance decisions, which can effectively avoid excessive maintenance or insufficient maintenance and thus reduce maintenance costs.

Currently, the prediction methods for equipment’s RUL can be divided into five categories: the prediction method based on state estimation, the prediction method based on the physical failure model, the prediction method based on experience, and prediction method based on evolution, and the prediction method based on data-driven feature propagation [2]. Among them, the data-driven method refers to the use of such methods as the neural network to extract the features and the relationship between the data from the state monitoring data of the equipment, so as to achieve the prediction of the equipment’s RUL. This is the application of artificial intelligence technology in fault prediction, which has more advantages than other methods. There have been many studies on this kind of prediction method. Chen et al. [3] realized fault detection and the diagnosis of high-speed trains by adopting a data-driven method. Meng et al. [4] used the variation trend of the degradation rate of monitoring parameters to predict the remaining life and analyzed the correlation between performance degradation failure and sudden failure. Ju et al. [5] studied a method to realize the fault prediction by using the improved support vector regression, which effectively improved the prediction accuracy of support vector regression algorithm for sudden failure. With the continuous development of deep learning in recent years, more and more deep learning models have been applied to the field of RUL prediction of equipment. Yuan et al. [6] realized the prediction of engine gas path faults by using convolutional neural network, and verified that the convolutional neural network is more feasible and effective than the traditional machine learning algorithm. Tang et al. [7] realized the prediction of the RUL of rolling bearings by using long short-term memory neural network (LSTM), which is suitable for processing long time series, and the method presented a good fitting effect. Zeng et al. [8] used a bidirectional LSTM o the predict airplane engine faults and obtained higher prediction accuracy compared with the recurrent neural networks (RNN), gate recurrent unit (GRU) and LSTM models. At present, the method of predicting the RUL of equipment is developing towards a hybrid model, and Ge et al. [9] applied t-distributed stochastic neighbor embedding (t-SNE) dimension reduction method to feature extraction and used LSTM for the RUL prediction of rotary machinery, which significantly improved the prediction and accuracy. Kang et al. [10] applied improved SAR and bidirectional LSTM to RUL prediction of rolling bearing, which improved the convergence speed of the model and obtained a lower prediction error. Song et al. [11] proposed an autoencoder–bidirectional LSTM (BLSTM) hybrid model to predict the turbofan engine RUL, and obtained better performance than the LSTM model.

Feature extraction has become one of the key problems in machine learning and data mining [12]. With the advent of the era of big data, the number of samples and features of data in many applications have increased dramatically, such as image classification and recognition, text classification, and fault detection. Such massive high-dimensional data are inevitably mixed with a lot of irrelevant and redundant information, which greatly affects the performance and efficiency of machine learning and deep learning algorithms. Therefore, when using high-dimensional data for deep learning tasks, feature extraction becomes very important. A large number of studies have shown that feature extraction can effectively reduce redundant and irrelevant information in the data, improve the efficiency of subsequent deep learning tasks, and improve the performance of the model. At present, the mainstream feature dimensionality reduction methods are divided into linear and nonlinear. Linear dimensionality reduction methods include principal component analysis (PCA) and linear discriminant analysis (LDA), which are often used to represent the overall interaction of the data [13]. The nonlinear dimension reduction methods include the kernel principal component analysis (KPCA), non-metric multidimensional scaling analysis (NMDS), isometric mapping, diffusion maps, and t-distributed stochastic neighbor embedding (t-SNE) are often used to express the local interaction of data [14]. Since the dimensionality reduction method in this work is only used for feature extraction, it is not suitable to select supervised learning methods that need label value. Moreover, the feature extraction method of this work should achieve three objectives: removing noise and redundant information, improving the accuracy of the model; reducing the computational complexity of the model, and improving the calculation efficiency; ensuring that the reduced dimension data can retain most of the useful information in the original data. Many of these methods are not suitable for use as feature extraction tools for this work. For example, LDA is a supervised learning method; kernel PCA, isometric mapping increases the computational complexity; and because the t-SNE target dimension needs to be less than 4, much information is lost.

Due to the close relationship between the variables of the status monitoring data of an airplane engine, if these features with a certain relationship can become new features that are unrelated, then the information of each variable in the original data can be reflected with fewer new features. Principal component analysis is such a dimensionality reduction method, which maps the original variables to the new space through the linear combination and constructs new variables to ensure that the new variables have no relationship with each other and retain the original information to the maximum extent. Then, the dimension of the new data is selected according to the variance contribution rate. PCA is a widely used unsupervised learning method with a solid mathematical foundation, which does not need label value and accords with the purpose of dimension reduction of this work. Therefore, PCA is chosen as the dimension reduction method. The results also show that PCA can improve the performance of the model.

Due to the high dimensionality of the status monitoring data of an airplane engine, the direct prediction may introduce useless information or noise, which reduces the accuracy of the prediction. This work chooses the method of PCA for airplane engine monitoring data dimension reduction, eliminating the useless monitoring information and noise, improving the generalization ability of the model. After the dimension reduction, the characteristic data are input into the BLSTM, and the advantage of BLSTM in processing long time series and the characteristics of forward and backward two-way propagation are utilized to obtain the relationship between the status monitoring data and RUL, so as to obtain more accurate prediction results.

## 2. Design of Hybrid Model based on PCA–BLSTM

The data of the airplane engine have the characteristics of a high dimension and a large amount of data, among which some useless information and noise affect the accuracy of the prediction model. Therefore, the feature extraction should be carried out first before the prediction to extract the most useful information. The feature extraction method adopted in this work is principal component analysis (PCA), which is fast, intuitive and can retain the original information to the greatest extent.

### 2.1. Principal Component Analysis

The idea of principal component analysis (PCA) [15] is to transform the original data into new data by using linear transformation, and the new data can retain the information of the original data to the maximum extent on the premise that each dimension is linearly independent. It is assumed that the original data have n dimensional feature vectors, which become K-dimensional vectors $Y_{1},Y_{2},\cdots Y_{k}$ after PCA feature extraction, as shown in Equation (1). $Y_{1}$ is the first principal component of the original variable after linear transformation, and similarly $Y_{1},Y_{2},\cdots ,Y_{k}$ are the second, third, and k principal components of the original data. Matrix A (as shown in Equation (2)) is the matrix composed of eigenvectors corresponding to the eigenvalue $\lambda_{i}$ of covariance matrix Σ (as shown in Equation (3)), where $a_{1},a_{2},\cdots a_{k}$ is the eigenvector corresponding to the eigenvalue $\lambda_{i}(i=1∼k)$:

(1) $$\left\{\begin{array}{c} Y_{1}=a_{11}X_{1}+a_{12}X_{2}+\cdots a_{1n}X_{n}\\ Y_{2}=a_{21}X_{1}+a_{22}X_{2}+\cdots a_{2n}X_{n}\\ \cdots \cdots \\ Y_{k}=a_{k1}X_{1}+a_{k2}X_{2}+\cdots a_{kn}X_{n} \end{array}\right.$$

(2) $$A=\left\{\begin{array}{c} a_{1}=a_{11}+a_{12}+\cdots a_{1n}\\ a_{2}=a_{21}+a_{22}+\cdots a_{2n}\\ \cdots \cdots \\ a_{k}=a_{k1}+a_{k2}+\cdots a_{kn} \end{array}\right.$$

(3) $$\sum =\sum_{i=1}^{n}\left(X_{i}-\bar{X}\right){\left(X_{i}-\bar{X}\right)}^{T}$$

### 2.2. BLSTM Neural Network

The BLSTM network [16] is composed of two directions of the LSTM layer. It can capture the information of the whole sequence from two directions during the training process of long time series, which has a higher performance than LSTM.

**Figure 1:**
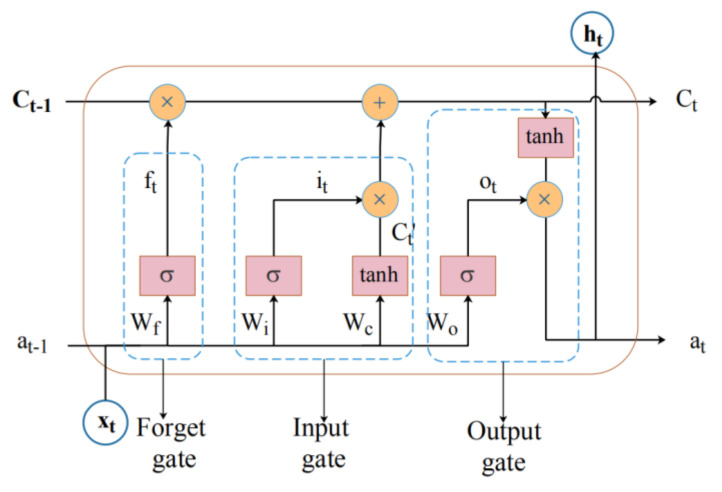
Long short-term memory neural network (LSTM) basic unit structure diagram.

LSTM networks are a variant of recurrent neural networks (RNN), which have appeared to solve the problem of gradient disappearance and gradient explosions that occur during the processing of long time sequences. The LSTM network extracts the internal relationship of long time series through the input gate, output gate and the forgetting gate, which is effective in dealing with a long-term dependence problem. Its basic unit structure diagram is shown in Figure 1.

As can be seen from the figure above, the stacked values of $a_{t-1}$ and $x_{t}$ are copied into four copies, and they are input into different doors. The details are described as follows:

In Figure 1, $i_{t}$, $f_{t}$ and $o_{t}$ respectively represent the operation results of the input gate, forget gate and output gate; $W_{f}$, $W_{i}$, $W_{c}$ and $W_{o}$ respectively represent the weight matrix of each part; b is the bias vector of each part; σ and tanh represent sigmoid function and hyperbolic tangent function respectively; $h_{t}$ represents for output; $C_{t}^{\prime }$ represents the candidate value of the current cell state; $C_{t}$ represents the updated cell status value.

The function of the forgetting gate is to choose how much of the state of the previous unit $C_{t-1}$ to retain or forget. The forgetting gate $f_{t}$ determines the influence of the old state information on the current unit. Its calculation formula is shown in (4):

(4) $$f_{t}=\sigma (W_{f}\times \left[a_{\left(t-1\right)},x_{t}\right]+b_{f})$$

The function of the input gate is to control the influence of the input on the current unit. It consists of two parts. Input $a_{t-1}$ and $x_{t}$ into sigmoid and tanh functions, respectively, to obtain the input information $i_{t}$ and the current cell state candidate value $C_{t}^{\prime }$. After multiplying the two values, and adding them to the historical state passing through the forgetting gate, the current state value $C_{t}$ of the unit is obtained. The calculation formula is as follows:

(5) $$i_{t}=\sigma (W_{i}\times \left[a_{\left(t-1\right)},x_{t}\right]+b_{i})$$

(6) $$C_{t}^{\prime }=\tanh(W_{c}\times \left[a_{\left(t-1\right)},x_{t}\right]+b_{c})$$

(7) $$C_{t}=f_{t}C_{t-1}+i_{t}C_{t}^{\prime }$$

The function of the output gate is to calculate the output value $a_{t}$. The output value is determined by two parts, one is the initial output value $o_{t}$ obtained by inputting $a_{t-1}$ and $x_{t}$ into sigmoid function, and the other is the state value $C_{t}$ after inputting the tanh function. Its calculation formula is as follows:

(8) $$o_{t}=\sigma (W_{o}\times \left[a_{\left(t-1\right)},x_{t}\right]+b_{o})$$

(9) $$a_{t}=h_{t}=o_{t}\times \tanh(C_{t})$$

**Figure 2:**
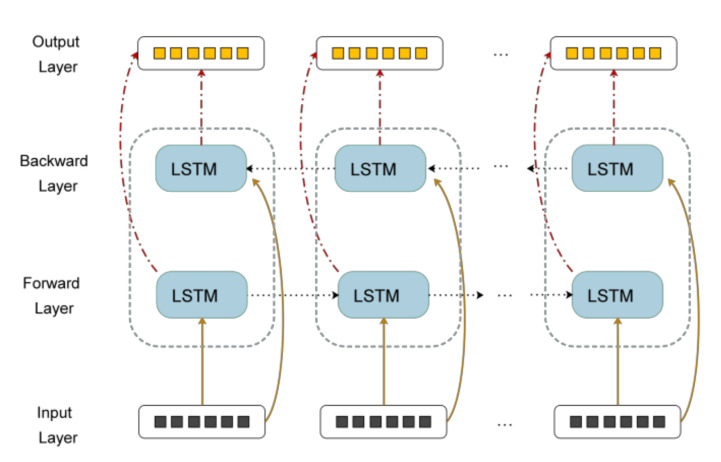
Bidirectional LSTM (BLSTM) structure diagram.

In the process of processing time series, the LSTM neural network can only use the historical information for forward propagation to predict the RUL of the device, ignoring the future information. BLSTM network can extract the internal relationship of the whole sequence from the two directions, so BLSTM is more suitable to be used as a prediction model. The basic idea of BLSTM is to connect the same input layer to both the forward and backward LSTM layers and combine the outputs of the two LSTMs to form the final output. In this way, the BLSTM layer can combine the historical and future information to make full use of the data. BLSTM can learn the internal relationship of the whole sequence, thus improving the performance of the model. The structure diagram of BLSTM is shown in Figure 2.

### 2.3. PCA–BLSTM Model Construction

**Figure 3:**
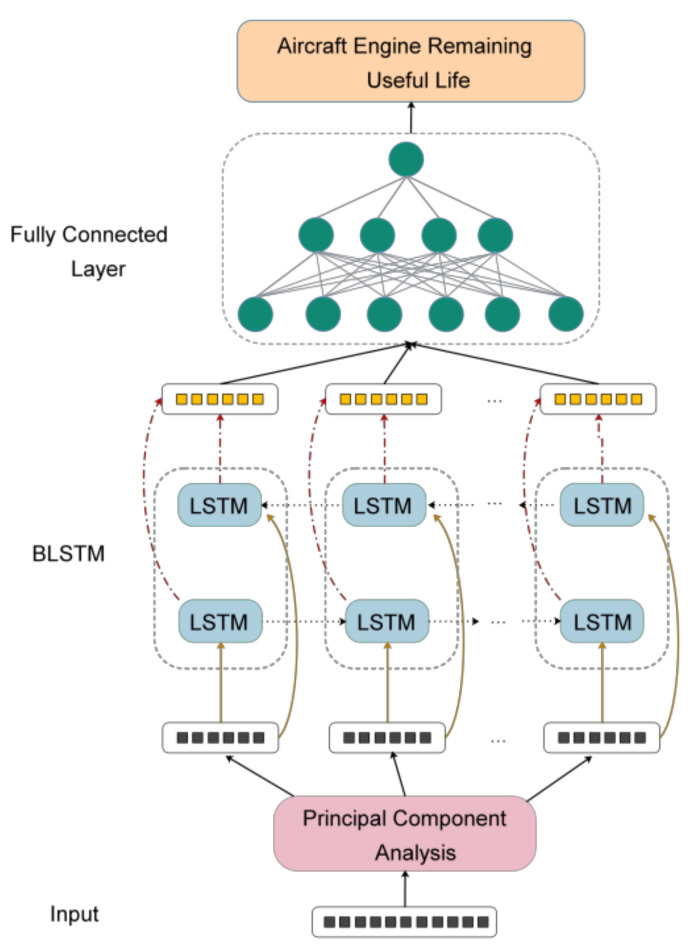
Structure diagram of the principal component analysis (PCA)–BLSTM hybrid model.

In this work, the hybrid model was adopted to conduct RUL prediction, and principal component analysis (PCA) was used as a dimensionality reduction tool to extract the characteristics of the status monitoring data of the airplane engine. After dimension reduction, the low-dimensional data was input into the BLSTM network for training. A two-layer BLSTM network was set up to mine deeper internal relationships of the data. However, this might result in overfitting. To prevent this phenomenon, the random dropout layer was added after each BLSTM layer and early stopping was used. At the end of the model, the full connection layer was added, the linear activation function was selected and the number of neurons was set to 1, so that the output of the hybrid model was consistent with the label. The structure of the PCA–BLSTM hybrid model is shown in Figure 3.

### 2.4. Training Process

**Figure 4:**
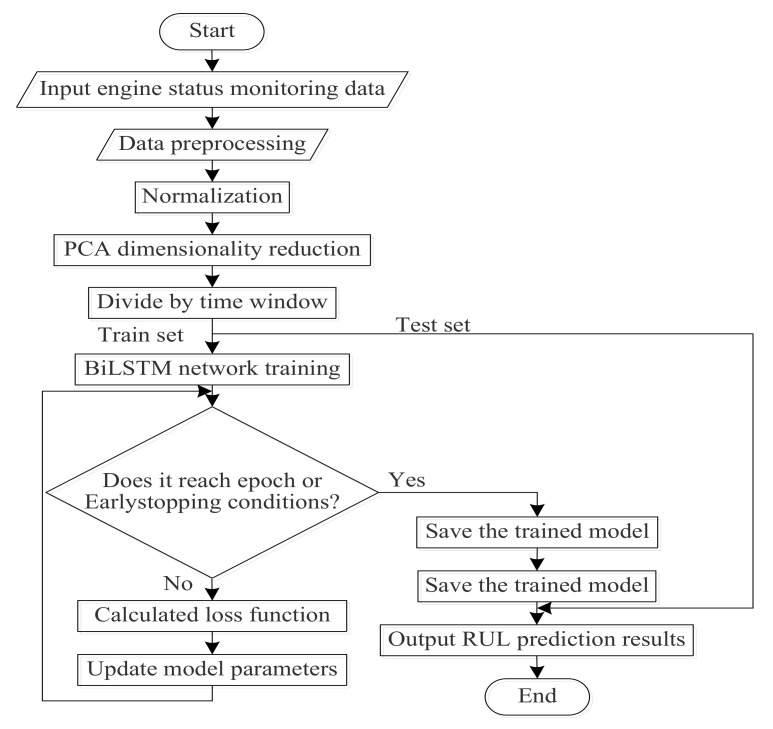
PCA–BLSTM model training flow chart.

**Figure 5:**
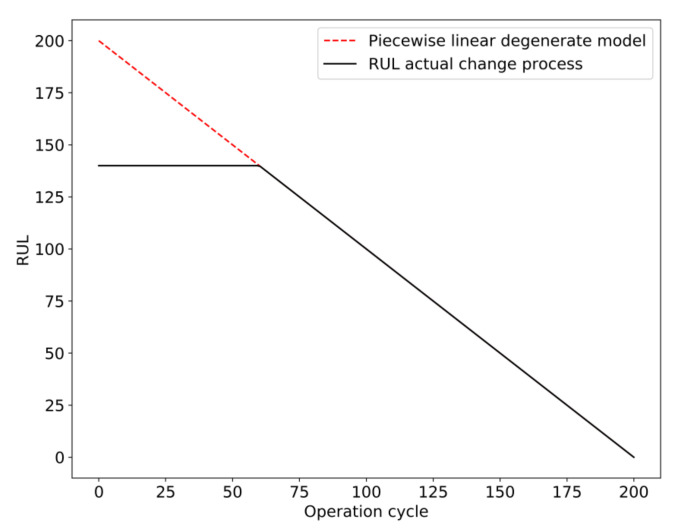
Piecewise linear degradation model.

The training flow chart of the PCA–BLSTM model is shown in Figure 4:

Before the prediction, the status monitoring data of the airplane engine were preprocessed and the RUL label was added. The engine failure process was a gradual process, so it is not suitable to use the actual RUL when adding the RUL label. Generally, the method adopted is to set the threshold of degradation and ignore the time before the engine degradation. After the operating time exceeds the threshold of degradation, the remaining useful life of the engine decreases monotonically. Piecewise linear degradation model [17] was proposed to solve this problem, as shown in Figure 5.

The status monitoring data of the airplane engine were composed of various sensor parameters of the airplane. In order to eliminate the difference of sensor parameter dimensions, each group of sensor parameters was linearly normalized, so that the parameter value was within the range of [0,1]. The formula is shown in (10):

(10) $$x^{\prime }=\frac{x-\min(x)}{\max(x)-\min(x)}$$

PCA dimensionality reduction was performed on the processed data, and the appropriate dimension was selected according to the size of the eigenvalue. Then, the training set and test set were segmented according to the size of the time step, and the segmented training set was input into the BLSTM model. The mean square error (MSE) was selected as the loss function, and its formula was shown in (11). Then, the loss function value of each bitch was calculated, root mean square prop (RMsprop) [18] was selected to optimize the loss function, the weight parameters of the model were adjusted, and the loss function value was minimized. The above operation was repeated when the maximum value of the epoch or early stopping condition was not reached. When any of these conditions were met, the trained model was saved. Finally, the test set after the PCA dimension reduction was input into the trained model and output the predicted RUL value:

(11) $$Loss=MSE=\frac{1}{n}{\sum_{i=1}^{n}\left(y_{i}^{\prime }-y_{i}\right)}^{2}$$

where $y_{i}^{\prime }$ represents the predicted value of the model and $y_{i}$ represents the label, and *n* is the number of samples.

## 3. Experimental Verification

### 3.1. Introduce NASAC-MAPSS

The data set adopted in this paper is from NASA’s Commercial Aerodynamic System Simulation (C-MAPSS) [19]. It is the benchmark data set in the field of RUL prediction of airplane engine. The C-MAPSS data set consists of four subsets, and each subset records the airplane engine health degradation data under different working conditions and failure types. In this paper, the FD003 subset was selected as the validation data set, and its basic information is shown in Table 1.

FD003 data set includes three working condition parameters: flight altitude, Mach number and throttle resolver angle. They can be combined into six working conditions that have a significant impact on engine performance. The data also include 21 engine sensors, such as the pressure at the fan inlet, burner fuel–air ratio and the bypass ratio [20]. The failure types of FD003 is the HPC degradation and fan degradation.

### 3.2. Data Set Validation

The airplane engine sensor parameters, working conditions parameters and the number of aircraft cycles are taken as training data, with a total of 25 dimensions. First, the original data $X=(X_{1},X_{2},\cdots X_{n})$ and the dimension K of the low-dimensional space are input, and then the original data are normalized. Then, calculating the covariance matrix $\sum =\sum_{i=1}^{n}\left(X_{i}-\bar{X}\right){\left(X_{i}-\bar{X}\right)}^{T}$; the eigenvalues of Σ and the corresponding eigenvectors are calculated, and the eigenvalues are sorted from the largest to the smallest. Then, all eigenvectors are normalized so that $a_{i}a_{i}{}^{T}=1(i=1∼n)$; then, the k eigenvectors are selected to form the matrix A; final output is $\left\{\begin{array}{c} Y_{1}=a_{11}X_{1}+a_{12}X_{2}+\cdots a_{1n}X_{n}\\ Y_{2}=a_{21}X_{1}+a_{22}X_{2}+\cdots a_{2n}X_{n}\\ \cdots \cdots \\ Y_{k}=a_{k1}X_{1}+a_{k2}X_{2}+\cdots a_{kn}X_{n} \end{array}\right.$.

The cumulative contribution rate of the eigenvalues of the covariance matrix is obtained as shown in Table 2.

The calculation formulas of the contribution rate and the cumulative contribution rate are as follows:

Formula of contribution rate:

(12) $$\frac{\lambda_{m}}{\sum_{k=1}^{n}\lambda_{k}}(m=1,2,\cdots n)$$

Formula of cumulative contribution rate:

(13) $$\frac{\sum_{m=1}^{n}\lambda_{m}}{\sum_{k=1}^{n}\lambda_{k}}(m=1,2,\cdots n)$$

Through the experiments, the prediction effect of the model is better when the target dimension is 12. After the dimension reduction, the time series of the training set and the test set are divided according to time step 50. The 3D tensor of the input model is (19720, 50, 12). Before the data are input into the model, various parameters of the model need to be set. Model parameters directly affect the performance of the model. Choosing appropriate parameters plays an important role in achieving a good prediction effect. After the experiment, the main parameters of the model were determined as shown in Table 3.

**Figure 6:**
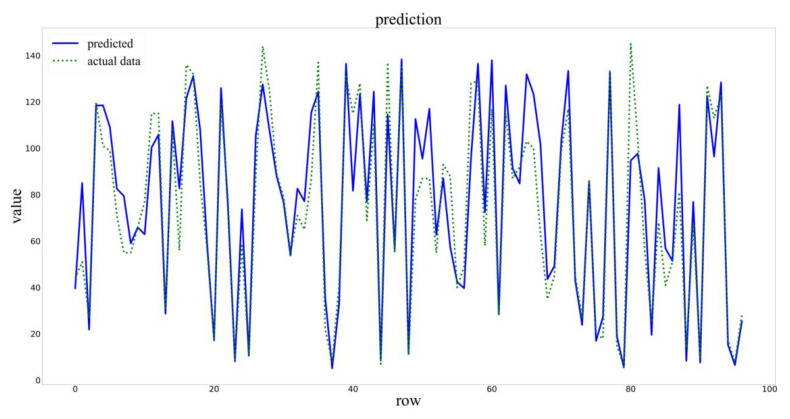
Prediction results of the PCA–BLSTM model.

The training set of the input model was divided into the training set and the verification set according to the ratio of 0.95:0.05. Setting the parameters of Early Stopping, if there was no improvement in the metrics of the verification set within 10 epochs, training would be stopped. Finally, the test sets were used to verify the accuracy of the model, and the predicted results were shown in Figure 6.

**Figure 7:**
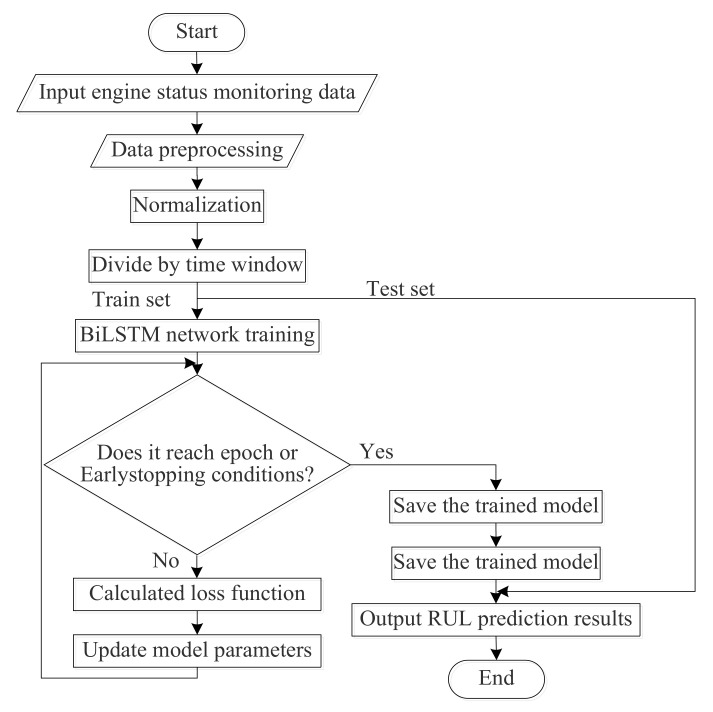
Training flow chart of the LSTM and BLSTM models.

To compare the model performance, the LSTM and BLSTM models were built. The training process is shown in Figure 7.

The data were also divided according to the time step 50, and various parameters of the model were set before the data were input into the model. The main parameters of the model were determined as shown in Table 4 and Table 5.

**Figure 8:**
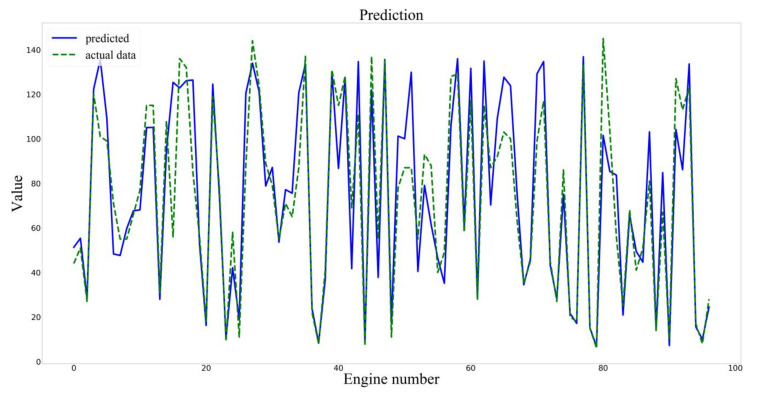
Prediction results of the LSTM model.

**Figure 9:**
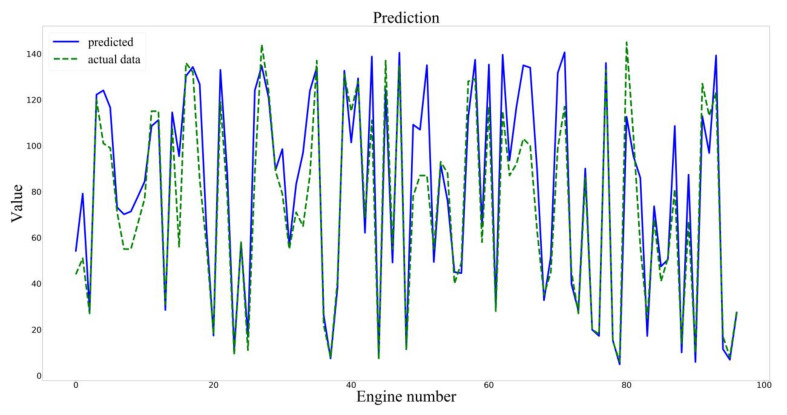
Prediction results of the BLSTM model.

The prediction results of the two models are shown in Figure 8 and Figure 9.

## 4. Comparison of Results

In the research on the remaining useful life of airplane engines, the evaluation criteria are mainly the root mean square error (RMSE) and the asymmetric scoring function to quantitatively evaluate the performance of models [21].

RMSE function can reflect the magnitude of prediction error, and its formula is as follows:

(14) $$RMSE=\sqrt{\frac{1}{n}{\sum_{i=1}^{n}\left(y_{i}^{\prime }-y_{i}\right)}^{2}}$$

where $y_{i}^{\prime }$ is the predicted value of the model, $y_{i}$ is the real value, and n is the number of samples.

If the RUL of the aircraft engine is overestimated, it will lead to inadequate maintenance, which will lead to serious consequences, so it is necessary to punish such a situation. Asymmetrical scoring functions produce higher penalties if the predicted value is greater than the true value. The score of asymmetric scoring function is inversely proportional to the performance of the model. That is, the lower the score, the better the performance of the model. The formula is shown in Equations (15) and (16):

(15) $$S=\frac{1}{n}\sum_{i=1}^{n}s_{i}$$

(16) $$s_{i}=\left\{\begin{array}{c} e^{-\frac{y_{i}^{\prime }-y_{i}}{13}}-1,y_{i}^{\prime }-y_{i}<0\\ e^{\frac{y_{i}^{\prime }-y_{i}}{10}}-1,y_{i}^{\prime }-y_{i}\geq 0 \end{array}\right.$$

where $y_{i}^{\prime }$ is the predicted value of the model, $y_{i}$ is the real value, and n is the number of samples.

The experimental results are compared with those of support vector regression (SVR), LSTM and BLSTM. Among them, in SVR, the linear kernel function was selected and the parameters were set as {C = 1, epsilon = 1}. LSTM uses double-layer LSTM for prediction and BLSTM consists of double-layer BLSTM and two-layer fully connected layer. Both models also use dropout and early stopping to prevent overfitting. The comparative test results are shown in Table 6.

The comparison of the test results shows that the PCA–BLSTM hybrid model is superior to the single model in both the error and score. The three deep learning methods have better performance than the machine learning method SVR. BLSTM has better performance than LSTM, which indicates that the BLSTM can combine historical and future information to fully explore the internal relationship of state monitoring data when processing long time series. The PCA–BLSTM hybrid model benefits from the use of PCA for feature extraction, which eliminates useless information and noise affecting the prediction accuracy and makes the model performed better. The PCA–BLSTM hybrid model proposed in this paper uses the data-driven method to predict the RUL of airplane engines, with high accuracy and no need to understand the engine structure and failure mechanism, as well as no need for professional knowledge and experience. It reduces the complexity of designing the model and can provide a decision basis for the maintenance and health management of aircraft engines.

## 5. Conclusions

In this paper, a hybrid model for predicting the RUL of an airplane engine based on PCA–BLSTM was proposed. Firstly, PCA is used to reduce the data dimension to extract features, and then combined with multi-layer BLSTM to extract the internal relationship of state monitoring data to achieve the RUL prediction. Compared with LSTM and BLSTM, the model shows a higher accuracy and better performance through the comparison of application results in the C-MAPSS data set, which can provide an intelligent decision basis for airplane engine maintenance and management. In the future, an attempt will be made to analyze the fault of the airplane engine, and a reasonable health management strategy will be proposed based on RUL prediction results.

## Figures and Tables

**Table 1:** FD002 data set details.

Name	Number of Engines in Training Set	Number of Engines in Test Set	Types of Working Conditions	Type of Failure	Number of Sensors	Type of Working Condition Parameters
FD003	100	100	1	2	21	3

**Table 2:** Eigenvalues and contribution rates.

Principal Component Sequence	Eigenvalue	Contribution Rate	Cumulative Contribution Rate
1	3659.51	0.3787	0.3787
2	2469.95	0.2556	0.6342
3	1264.72	0.1309	0.7651
4	616.37	0.0638	0.8289
5	402.09	0.0416	0.8705
6	190.44	0.0197	0.8902
7	174.23	0.018	0.9082
8	165.91	0.0172	0.9254
9	141.84	0.0147	0.94
10	116.44	0.012	0.9521
11	93.17	0.0096	0.9617
12	89.98	0.0093	0.971
13	83.77	0.0087	0.9797
14	76.8	0.0079	0.9877
15	61.47	0.0064	0.994
16	35.34	0.0037	0.9977
17	7.79	0.0008	0.9985
18	7.42	0.0008	0.9992
19	7.3	0.0008	1

**Table 3:** Parameter setting of PCA–BLSTM hybrid model.

Parameter	Value
Degradation threshold	140
Units in the first layer of BLSTM	100
Units in the second layer of BLSTM	100
Units in the first layer of the full connection layer	30
Units on the second layer of the full connection layer	1
Dropout	0.2
Bitch	100

**Table 4:** Parameter setting of the LSTM hybrid model.

Parameter	Value
Degradation threshold	140
Units in the first layer of BLSTM	100
Units in the second layer of BLSTM	50
Units of the full connection layer	1
Dropout	0.2
Bitch	200

**Table 5:** Parameter setting of the BLSTM hybrid model.

Parameter	Value
Degradation threshold	140
Units in the first layer of BLSTM	100
Units in the second layer of BLSTM	50
Units in the first layer of the full connection layer	30
Units on the second layer of the full connection layer	1
Dropout	0.2
Bitch	100

**Table 6:** Comparison of the experimental results.

Model	RMSE	Score
SVR	25.69	52.84
LSTM	11.99	15.22
BLSTM	11.65	6.69
PCA–BLSTM	11.1	4.49
